# A Sleep Sensor Made with Electret Condenser Microphones

**DOI:** 10.3390/clockssleep7020028

**Published:** 2025-05-31

**Authors:** Teru Kamogashira, Tatsuya Yamasoba, Shu Kikuta, Kenji Kondo

**Affiliations:** 1 Department of Otolaryngology and Head and Neck Surgery, Faculty of Medicine, University of Tokyo, Tokyo 113-8655, Japan; kondok-tky@umin.ac.jp; 2 Department of Otolaryngology, Tokyo Teishin Hospital, Tokyo 102-8798, Japan; tyamasoba-tky@umin.ac.jp; 3 Department of Otolaryngology and Head and Neck Surgery, Nihon University, Tokyo 173-8610, Japan; kikuta.shu@nihon-u.ac.jp

**Keywords:** flow measurement, home sleep apnea test, obstructive sleep apnea, electret condenser microphone, sleep-disordered breathing, infrasound, extremely low frequency

## Abstract

Measurement of respiratory patterns during sleep plays a critical role in assessing sleep quality and diagnosing sleep disorders such as sleep apnea syndrome, which is associated with many adverse health outcomes, including cardiovascular disease, diabetes, and cognitive impairments. Traditional methods for measuring breathing often rely on expensive and complex sensors, such as polysomnography equipment, which can be cumbersome and costly and are typically confined to clinical settings. These factors limit the performance of respiratory monitoring in routine settings and prevent convenient and extensive screening. Recognizing the need for accessible and cost-effective solutions, we developed a portable sleep sensor that uses an electret condenser microphone (ECM), which is inexpensive and easy to obtain, to measure nasal airflows. Constant current circuits that bias the ECM and circuit constants suitable for measurement enable special uses of the ECM. Furthermore, data transmission through the XBee wireless communication module, which employs the ZigBee short-range wireless communication standard, enables highly portable measurements. This customized configuration allows the ECM to detect subtle changes in airflow associated with breathing patterns, enabling the monitoring of respiratory activity with minimal invasiveness and complexity. Furthermore, the wireless module not only reduces the size and weight of the device, but also facilitates continuous data collection during sleep without disturbing user comfort. This portable wireless sensor runs on batteries, providing approximately 50 h of uptime, a ±50 Pa pressure range, and 20 Hz real-time sampling. Our portable sleep sensor is a practical and efficient solution for respiratory monitoring outside of the traditional clinical setting.

## 1. Introduction

Measurement of nasal airflow during sleep is essential to evaluate sleep quality [1] and detect sleep apnea syndrome [2]. Specifically, nasal airflow monitoring is useful to detect sleep-related breathing disorders such as sleep apnea [3], which is characterized by repetitive interruptions in breathing. Such interruptions in breathing can lead to fragmented sleep, reduced oxygen levels, and increased risks of serious health problems such as cardiovascular disease [4], diabetes [5], and cognitive impairments [6]. Early and accurate detection of these conditions through respiratory measurements is important to support timely intervention and treatment. Tracking airflow through the nostrils can provide valuable insight into an individual’s breathing stability and overall sleep quality, which is crucial for early diagnosis and treatment planning for such disorders. However, expensive sensors are required to measure nasal airflow, and thus, an inexpensive sensor that can measure airflow and evaluate breathing conditions needs to be developed. We have developed a portable sleep sensor using an electret condenser microphone (ECM) as a micro-pressure sensor. The ECM can be utilized by constructing a circuit with bias and circuit constants suitable for the measurement. By using the ECM as a low-frequency pressure sensor for airflow detection [7,8], nasal airflow can be measured simply and inexpensively. Furthermore, the ECM is highly portable due to the achievement of data transmission using the ZigBee short-range wireless communication standard.

## 2. Results

**Figure 1:**
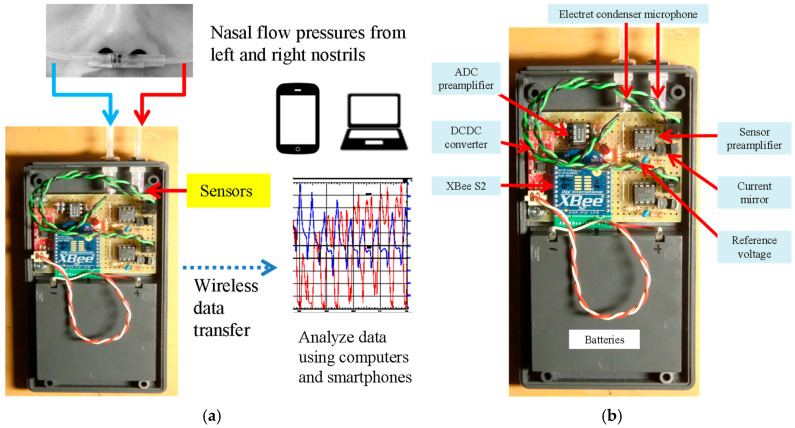
Sleep sensor. (**a**) Overall system. (**b**) Portable sensor.

The sensor specifications are shown in Table 1, and an overall view is shown in Figure 1. Tubes that detect nasal airflows are commonly available as nasal cannulas and are connected to an ECM through the attachment of the sensor box.

**Figure 2:**
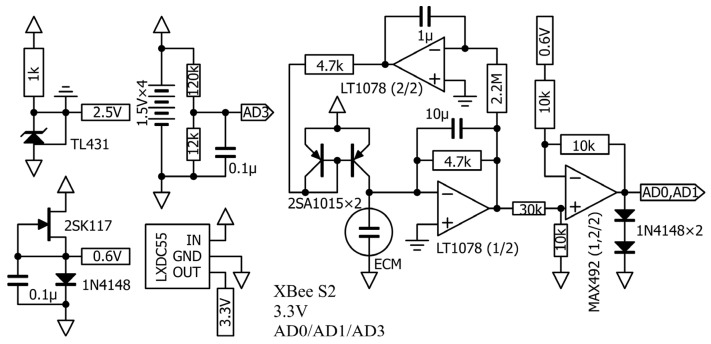
Diagram of the overall circuit.

The overall circuit diagram is shown in Figure 2. Details of the equipment components are provided in Appendix A. Data are received by the XBee parent unit, and the results are sequentially recorded in a file by a display and recording program using Processing software (Appendix B).

**Figure 3:**
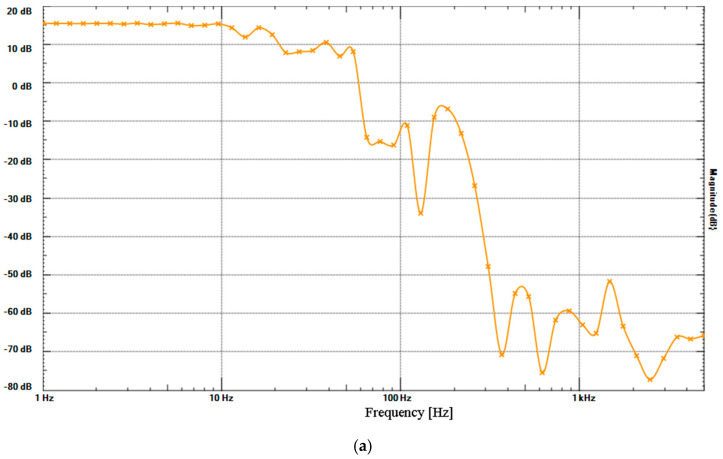

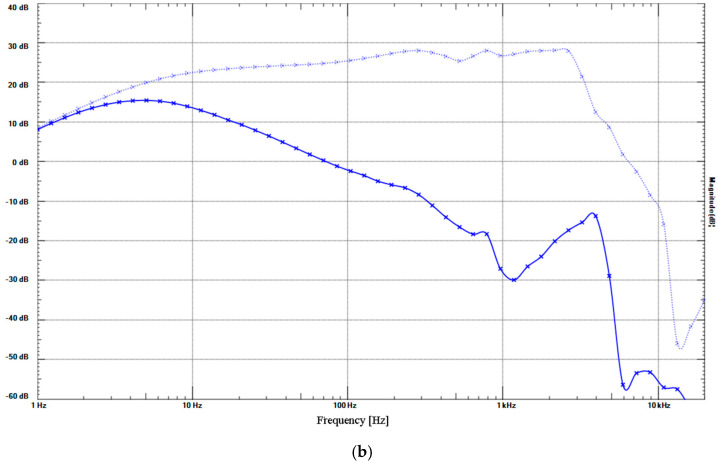
Frequency responses of pressure generators and sensors before the XBee analog-to-digital converter (ADC) measured with Analog Discovery (Digilent Inc., Austin, TX, USA). (**a**) Frequency responses of micro-pressure generator with a micro-speaker measured with GC30-101-C9N380 ±100 Pa. (**b**) Frequency responses of micro-pressure generator with a micro-speaker measured with an ECM using a circuit shown in Figure 2—solid line, with LPF (3.4 Hz; 4.7 kΩ, 10 μF); dotted line, without LPF; *x*-axis, frequency [Hz]; *y*-axis, magnitude [dB].

The digital micro-differential pressure gauge (GC30-101-C9N380 ±100 Pa; Nagano Keiki Co., Ltd., Tokyo, Japan; registered under the Japan Calibration Service System (JCSS) and compliant with the international Mutual Recognition Arrangement (MRA)), exhibited an almost flat frequency response from near direct current (DC) to 10 Hz when the sampling rate was 20 Hz, without an anti-aliasing filter (Figure 3). In the measurement of the ECM, the frequency response without an low-pass filter (LPF) (omitting the 10 μF capacitor of the transimpedance amplifier) was 6 dB/oct high-pass filter (HPF), with a cutoff frequency of around 10 Hz, which is a higher frequency than the 0.07 Hz frequency of the DC servo (Figure 3). When the LPF of 3.4 Hz was set in the transimpedance amplifier, the frequency response of the band-pass filter (BPF) was about 5 Hz. Based on the frequency response curve, the resonant frequency of the micro-speaker of the micro-pressure generator was around 2 kHz. There was no response up to DC because the ECM was not tightly sealed to the diaphragm. Although it is possible to perform simple measurements of ultra-low frequency noise, it might be difficult to measure infrasound.

**Figure 4:**
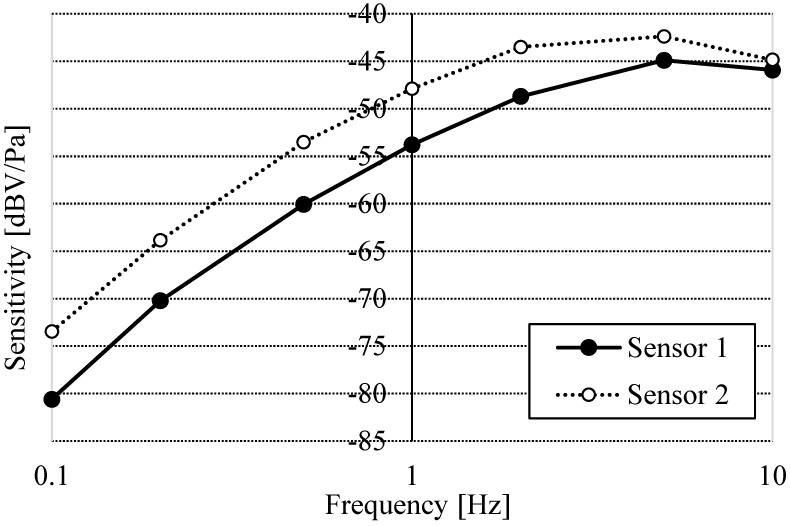
Calibration of sensitivity with multiple sensors using the XBee ADC using a circuit shown in Figure 2. *x*-axis: frequency [Hz]; *y*-axis: sensitivity [dBV/Pa].

An example of a calibration using the XBee analog-to-digital converter (ADC) is shown in Figure 4. There were sensitivity differences, depending on the ECM, in the frequency region below 10 Hz, so calibration before measurement is required to compensate for gain differences.

**Figure 5:**
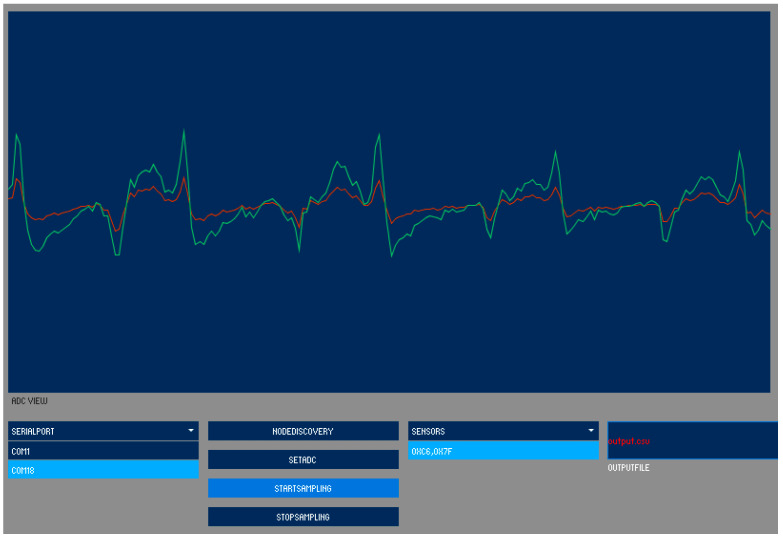
A display sample of measurement of nasal airflows in a recording program using Processing software. Graphs are for confirmation only, and the detailed unit display is omitted.

**Figure 6:**
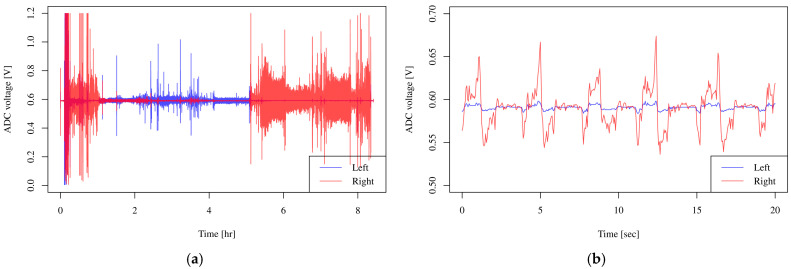
The measurement sample raw data. (**a**) Long-term recording results of overnight sleep. (**b**) Data for 30 s from 20 min after the start of recording in (**a**). Values in the positive direction from the center indicate an expiratory phase, and values in the negative direction indicate an inspiratory phase.

When the sensor is turned on, it will automatically start looking for the XBee parent unit and automatically connect to it. Nasal airflows can be monitored and recorded by starting a measurement program using the Processing software on the computer connected to the XBee parent unit, registering the sensor unit, and starting recording. Airflow measurement data are displayed on the screen for a certain period of time and are also saved in a text file, which can be analyzed later (Figure 5). Figure 6a shows the results of overnight respiratory monitoring. In the magnified trace (Figure 6b), positive values from the center indicate the expiratory phase, while negative values indicate the inspiratory phase. A difference in airflow between the left and right nasal passages was observed, reflecting the nasal cycle associated with body movements such as turning over during sleep. In the prototype, the average current consumption was 37.4 mA, which was within the design specifications, and the device was considered capable of operating for more than two days.

## 3. Discussion

In this report, we propose a portable nasal airflow meter system that uses an ECM as a micro-pressure sensor. The device is easy to produce and inexpensive. By measuring airflow over time, this system is expected to be useful for the detection of sleep apnea syndrome [2]. It is also expected to facilitate the evaluation of sleep quality [1]. In addition, by performing double recordings of nasal airflow, it is possible to monitor the nasal cycle [9,10], which could yield information regarding the functioning of the autonomic nervous system [11,12].

**Figure 7:**
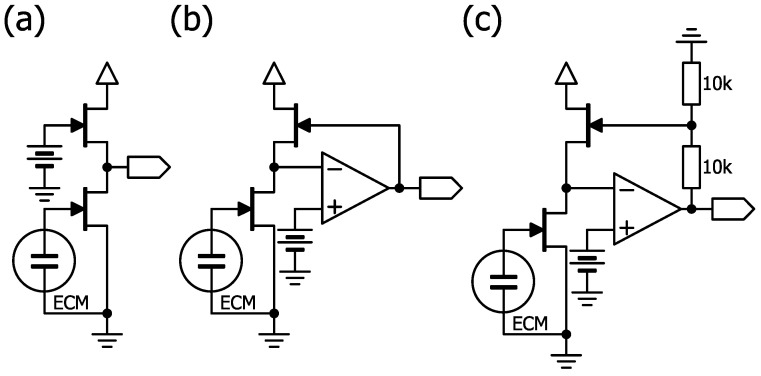
Circuit examples for ECM bias using a cascode-like circuit. These configurations represents a theoretical design concept and has not yet been experimentally validated. (**a**) Simple cascade-like circuit. (**b**) Cascode-like circuit with operational amplifier. (**c**) Cascode-like circuit using operational amplifier with gain (×2).

Although distortion does not affect simple nasal airflow measurement, a considerable amount of secondary distortion occurs when the signal is large due to the square characteristic of continuous drain current (Id) − gate-source voltage (Vgs). To solve this problem, another circuit (Figure 7) has been proposed that cancels the square characteristic. The gain and offset voltage are strongly dependent on pinch-off voltage (Vpo) and drain-source leakage current (Idss); however, in this system, it is theoretically possible to handle large signals with low distortion by selecting an appropriate constant-current FET. Our primary focus was to establish the feasibility of the proposed constant-current bias circuit in terms of improving the power supply rejection ratio (PSRR) through theoretical analysis; as such, no experimental measurements were conducted in this study, and future work will aim to validate the design empirically and compare its performance with that of alternative circuits.

To detect respiratory waveforms, special sensors such as thermistors [13], expensive piezoelectric films [14], and strain gauge pressure sensors [15] are required [16,17] because the respiratory frequency is less than 1 Hz. Recently developed flexible and stretchable self-powered sensors [18] are cost-effective and can be utilized for this purpose in applications where pressure data are not required. In a recently reported system for the non-invasive measurement of nasal airflow velocity, a temperature sensor and wireless data transmission were used [19]; however, this system is inferior to those using pressure sensors in terms of measurement accuracy [13,20]. To evaluate the clinical usefulness of the ECM pressure measurements, it will be important to validate them against a gold-standard reference (pneumotachograph) in a clinical trial. In addition, pressure sensors have the advantage of being able to detect more apnea events than thermistors in polysomnography [21,22]. Our system uses an inexpensive and readily available ECM as a low-frequency pressure sensor for the simple and highly accurate measurement of nasal airflow. In addition, data can be transmitted using an XBee wireless module that implements the ZigBee short-range wireless communication standard [23,24,25], enabling highly portable measurements. This simple and highly accurate system could also be applied to other fields, such as measuring minute pressures inside bronchi [26].

Future developments include implementing algorithms to address artifacts commonly encountered in home sleep monitoring—such as body movement and mouth breathing—and comparing the false-positive and false-negative rates of the portable sleep sensor with those of polysomnography.

As respiratory monitoring technologies continue to advance for use in more accessible and non-clinical settings, parallel progress in neural interface research is redefining how humans engage with technology and their surroundings. Neural interfaces—which connect the brain to external devices by decoding neural signals—hold promise not only in healthcare but also in broader societal contexts. However, their development is hindered by persistent challenges, including material–tissue compatibility, signal fidelity, and algorithmic limitations. These obstacles, as emphasized in recent discussions on the state of neural interface research, highlight the need for interdisciplinary approaches to develop effective, adaptable brain–computer interaction systems [27].

## 4. Materials and Methods

### 4.1. Sensor and Circuit Overview

This study employed the XCM6035-2022-354R (SPL Limited, Kowloon, Hong Kong) ECM, which is nearly equivalent to the WM-61A (Panasonic Semiconductor/Nuvoton Technology Corporation, Kyoto, Japan). To suppress left–right crosstalk, the preamplifier stage for the signal from the ECM is a transimpedance amplifier using a current mirror. To suppress high-frequency signals from the ECM and reduce power consumption, the gain bandwidth product (GBWP) of the operational amplifier (op-amp) is 500 kHz or less, and a high-precision type with a low input offset voltage was selected so that offset adjustment is not required to simplify the circuit.

In this example, an LT1078 (Linear Technology Corporation/Analog Devices, Wilmington, MA, USA) device was used in the microphone preamplifier stage, while a MAX492 (Maxim Integrated Products/Analog Devices, Wilmington, MA, USA) device, with rail-to-rail operation at the input and output, was used in the XBee ADC preamplifier stage. The input of the XBee Series 2 ADC ranges from 0 V to 1.2 V, meaning that the diode is driven with a constant current to generate a midpoint voltage of about 0.6 V, which is then converted at the preamplifier stage and input to the XBee ADC.

### 4.2. Wireless Data Transmission Module

To allow for 2 days of operation, the power consumption requirements of the wireless module are important in its selection. Table 2 shows wireless modules that were available at the time of the study.

The power source of this sensor is four AA batteries, connected in series. Based on the datasheet of Panasonic’s website (Appendix C), a module with a current consumption of 40 mA or less must be used for AA batteries to sustain the operation for more than 2 days. If a switching regulator is used for stepping down, the efficiency will be higher, and thus, the required conditions will be more lenient. All modules using standard Wi-Fi are unsuitable for this sensor because they consume more than twice the power of 40 mA. There was no Java software development kit (Java SDK) available for TWE-Lite. Modules using Bluetooth Low Energy (Bluetooth LE) consume little power and are suitable for this sensor, but are incompatible with Windows 8. There were many issues with interoperability and SDKs in using Bluetooth LE, and earlier versions of Processing software do not support the API library for Processing (Java); therefore, the XBee S2 module was adopted. Once the PAN ID of the coordinator is set on the modules of XBee, they will automatically search for and connect to the network just by turning the device on, making them very easy to use.

### 4.3. Consideration of the Sensor Amplifier Circuit

**Figure 8:**
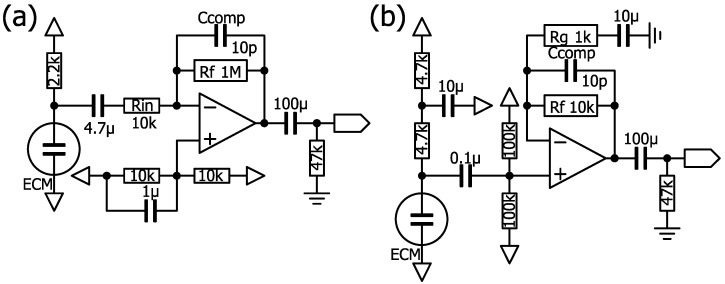
Circuit examples of the ECM. (**a**) Inverting amplifier circuit. (**b**) Non-inverting amplifier circuit.

The typical circuits that use op-amps to amplify ECM signals with a single power supply, such as batteries, in portable devices are an inverting amplifier circuit and a non-inverting amplifier circuit (Figure 8). The problem with these circuits is that the ECM is directly connected to the power supply through a load resistor of several kΩ, and the midpoint is biased with two resistors and a capacitor, which causes crosstalk between multiple channels operated with a single power supply. Another problem with these circuits is that the output voltage saturates at high sound pressures of about 100 dB SPL or higher in nasal airflows. The requirements for a circuit to detect nasal airflows are low crosstalk and tolerance to high sound pressures.

### 4.4. Possibility of Reducing Crosstalk Using Regulators

One possible idea to solve the crosstalk problem is to use a constant voltage regulator in each channel. As an experiment, employing a low noise 3.3 V regulator (NJM2863, Nisshinbo Micro Devices Inc., Tokyo, Japan) as a supply voltage to both channels reduced the crosstalk, but series regulators for the constant voltage circuit in battery-powered mode can be problematic in terms of power consumption. Another problem in measuring at high sound pressure using regulators is that the output voltage fluctuates and is difficult to stabilize after the input of high sound pressure. The specified power supply voltage is low at about 2 V, and consequently, the signal from the ECM saturates when the signal is large, causing the bias voltage to fluctuate. To solve this problem when the signal is large, modification of the internal circuit of the ECM itself from an original common source circuit to a common drain (source follower) has been proposed by some enthusiasts. However, this method is difficult and requires an accuracy of less than 1 mm when cutting the terminals of the ECM to separate the source and ground.

### 4.5. Biasing an ECM with a Constant Current

**Figure 9:**
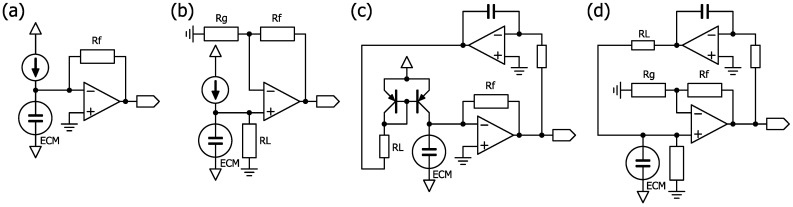
Circuit examples for ECM bias. (**a**) Constant current bias with I/V conversion by an op-amp. (**b**) Constant current bias with a non-inverting amplifier circuit. (**c**) I/V conversion with servo by an op-amp, as described in the LT1677 datasheet. (**d**) Non-inverting amplifier circuit with servo.

The operating point of the ECM can be controlled by replacing the load resistance with a constant current load, allowing the ECM to be biased at a higher voltage. High voltage bias increases output impedance but also increases amplification. The typical circuits for this bias method, using current/voltage (I/V) conversion with an op-amp or amplifying with a non-inverting amplifier circuit, are shown in Figure 9. The circuit constant of a constant current should be determined based on the maximum current consumption of the field-effect transistor (FET) specified in the datasheet (usually about 0.5 mA) used in the ECM (2SK3372 (WM-61A), maximum Idss: 460 μA). Adjustment of the constant current is necessary when the output sticks to the power supply voltage or ground, depending on the operating point. In the case of a non-inverting amplifier circuit, the ECM is biased through a load resistor from the servo circuit (Figure 9). An I/V conversion circuit with a servo was adopted to allow for flexibility in circuit constants.

### 4.6. Consideration of Circuit Constants and Components

#### 4.6.1. Power Supply Voltage

If two AA batteries (3 V) are used, the step-up converter to supply 3.3 V power to a wireless module may be less efficient in obtaining the sufficient current. Additionally, due to the ease of designing circuits using a single power supply or rail-to-rail op-amp, as well as the type of battery case available, four AA batteries were adopted.

#### 4.6.2. I/V Resistance

The sensitivity of XCM6035-2022-354R, which is nearly equivalent to that of WM-61A, is −35 dBV, and the voltage at 1 Pa (94 dB SPL) is 10^−35/20^ = 17.8 mVrms. The specified resistance is 2.2 kΩ; therefore, the current is 17.8 mV/2.2 kΩ = 8.1 μArms/Pa. The maximum sound pressure of the ECM itself is not specified, but the tube pressure measurement evaluates a relatively high pressure as sound pressure. Therefore, to ensure that the peak-to-peak voltage after I/V conversion at 128 dB SPL (50 Pa) was within 6 V (the equivalent of four 1.5 V dry batteries), the I/V resistance is 6 V/(50 × 8.1 × 2 × sqrt(2)) μApp = 5.2 kΩ, and 4.7 kΩ was selected from the E series as a slightly smaller value, with some leaks.

#### 4.6.3. Low-Pass Filter (LPF) and High-Pass Filter (HPF)

The breathing rate varies from 10 to 20 breaths per minute, but the measurement range is slightly lower than this frequency. The LPF of the transimpedance amplifier was set to 3.4 Hz (4.7 kΩ, 10 μF), while the DC servo (HPF) was set to 0.07 Hz (2.2 MΩ, 1 μF). This frequency is low enough to preserve the relevant low-frequency components of the respiratory signal.

#### 4.6.4. Reference Voltage

The shunt regulator TL431 was selected for a reference voltage because it was readily available, but other types can be adopted from the perspective of power consumption. TL431 requires a minimum current of 1 mA, according to the datasheet. In the worst case scenario, the voltage of four AA batteries connected in series, with voltage drop, is 4 V, and the resistance should be 1.5 kΩ or less; therefore, 1 kΩ was selected to flow 1 mA or more at 4 V − 2.5 V = 1.5 V. To extend the operating time, NJM2825 (Nisshinbo Micro Devices Inc., Tokyo, Japan; minimum current: 0.7 μA), LT1389 (Linear Technology Corporation/Analog Devices, Wilmington, MA, USA; minimum current: 0.7 μA), or similar components can be adopted. The output current limitation should be checked to conform to the upper limit of the input bias current of the op-amp.

#### 4.6.5. XBee ADC Midpoint Voltage

In XBee Series 2 and later versions, the maximum input voltage is fixed at 1.2 V, unlike XBee Series 1 and XBee Pro with programmable HCS08, in which the reference voltage for the ADC (Vref) can be set. The forward drop voltage (VF) of the diode was utilized to set the midpoint (0.6 V) with a maximum input of 1.2 V for the ADC. The diode was biased with a constant current using a FET (2SK117, Idss: 1.2–3.0 mA (Y)) because of the simplicity of the circuit. When temperature characteristics, voltage accuracy, and power savings are required, TL431 can also be adopted.

#### 4.6.6. Resistor to Measure Remaining Battery Capacity

Voltage precision is not required; therefore, the division using 120 kΩ and 12 kΩ was utilized to directly input to the XBee ADC (input impedance: 10 kΩ). Current consumption is about 45 μA, which is much less than that of the wireless module and does not pose a problem in terms of battery operating time.

#### 4.6.7. Op-amps

##### Requirements for Op-amps to Amplify the ECM

The maximum voltage of four AA batteries is 1.6 V × 4 = 6.4 V, and is 7 V with a margin. The package is a plastic dual in-line package (PDIP), employed to easily handle components. The I/V resistor is 4.7 kΩ; therefore, the input bias current of the op-amp should be 0.2 μA or less when the error is required to be 1 mV or less. The input offset current also affects the error to the same extent as the feedback resistor; therefore, this parameter is also set to the same condition. The amplification factor in this circuit is low, and the input offset voltage does not severely affect the error. When the ADC range is 1.2 Vpp and the error is kept within ±1 bit with a 12-bit ADC, the offset voltage should be within 1.2 V/2^12^ × 2 = 0.6 mV. The offset voltage of a typical op-amp is about 1 mV, which generally satisfies this condition. The frequency to be handled is 10 Hz or less; therefore, a GBWP of several hundred kilohertz or less is sufficient, and a high-speed op-amp that consumes power is unnecessary. The sink current must be 1 mA or more to bias the load resistor connected to the current mirror and to supply power to the ECM (about 0.5 mA).

In summary, the conditions are as follows: (1) two channels, 8-pin PDIP, maximum voltage of 7 V or more, single power supply-compatible, rail-to-rail or output up to about 1 V below the power supply voltage; (2) as little current consumption as possible; (3) input bias current and input offset current of 0.2 μA or less; (4) input offset voltage of 1 mV or less, (5) GBWP of several hundred kilohertz or less; and (6) maximum output current of 1 mA or more.

An example of results based on the aforementioned conditions using an op-amp search system provided by a semiconductor manufacturer is shown in Appendix D. LT1078 was selected from the list based on its availability.

##### Op-amp Current

Op-amps with a large GBWP and large slew rate consume a lot of power and are unsuitable for this purpose, but several op-amps were tested for proper operation. NJM2732 (Nisshinbo Micro Devices Inc., Tokyo, Japan) and LMC6482 (National Semiconductor/Texas Instruments, Dallas, TX, USA) worked normally, but the output was stuck to the power supply voltage or ground with NJU7096 (Nisshinbo Micro Devices Inc., Tokyo, Japan). The maximum source/sink current is about 10/2 and 30/20 mA for NJM2732 and LMC6482, respectively, which is greater than the maximum current of the ECM. However, the maximum source/sink current for NJU7096 is only about 200/1000 μA, which is insufficient to supply power to the ECM.

##### Op-amp Noise

The frequency band in this circuit is relatively narrow (0.07–3.4 Hz); therefore, noise is unlikely to be a problem. The estimated noise based on the LT1078 datasheet is detailed below. The input-equivalent noise current density is 0.06 pA/sqrt(Hz)@10 Hz, and 1/f noise from 0.07 Hz to 3.4 Hz is 0.06 pA × sqrt(10 × ln(3.4/0.07)) = 0.24 pArms, which is 1.1 nVrms when the feedback resistor is 4.7 kΩ. The input-equivalent noise voltage density is 29 nV/sqrt(Hz)@10 Hz with a 1/f corner of 0.7 Hz. The graph in the datasheet shows that in this region, where 1/f noise is not dominant, the band noise is 30 × sqrt(3.4 − 0.07) = 55 nVrms, which will not affect the measurements.

##### Level Shifting of the Op-amp

The conditions of the preamplifier op-amp, which performs level shifting from 2.5 V to 0.6 V in order to connect to XBee ADC, are more lenient than those described above. The output voltage is low at 0–1.2 V; therefore, MAX492, which is a rail-to-rail low-power op-amp, was selected.

### 4.7. Pressure Calibration Using a Micro-Pressure Sensor

The lower limit of the ECM is generally up to about 50 Hz in the frequency response figure provided in the datasheet (Appendix E); therefore, the frequency response below 50 Hz was measured. Air pressure handled by this sensor is very low at several hundred pascals, which is difficult to measure with a general barometer. A small digital micro-differential pressure gauge (GC30) was used to measure the frequency response of the pressure to the ECM. A calibration device to generate a very small pressure with a sine wave for this measurement was also produced (Appendix F).

## 5. Conclusions

This work presents a portable nasal flow-measuring system that uses an ECM as a micro-pressure sensor, making it simple and inexpensive to fabricate the device. This portable sleep sensor provides a practical, accessible, and cost-effective solution to measure nasal airflow during sleep. The circuit biasing the ECM and the suitable circuit constants for measurements enable the specific use of the ECM. In addition, data transmission using the ZigBee short-range wireless communication standard enables the achievement of highly portable measurements. The sensor provides a convenient way to assess sleep quality and detect potential sleep disturbances and can improve the accessibility of sleep monitoring, while maintaining high portability and ease of use.

## 6. Patents

This work is patented as PCT/JP2019/005287 (WO/2019/167643) “Rhinomanometry Device”.

## Figures and Tables

**Table 1:** Main specifications of the sensor.

Power supply	Four AA batteries (1.5 V)
Uptime	About 2 days (50 h)
Sensing pressure	±50 Pa
Sensor response frequency	Around 3 Hz
Sampling Rate	20 Hz
Data output	Wireless/Real-time

**Table 2:** Power consumption of wireless modules.

Module	Standard	Current Consumption	Remarks
XBee S2	ZigBee	Transmit: 33 mA Receive: 28 mA	Java SDK is available.
TWE-Lite (TWE-001L)	IEEE802.15.4	Transmit: 17 mA Receive: 15 mA	Java SDK is not available.
XBee S6 (Wi-Fi)	Wi-Fi	Transmit: 309 mA Receive: 100 mA	
ESP8266 (ESP-WROOM-02)	Wi-Fi	Average 80 mA	
ESP32 (ESP-WROOM-32)	Wi-Fi + Bluetooth LE	Wi-Fi transmission 160–260 mA	
nRF51822	Bluetooth LE	Transmit: 16 mA	Requires Windows 8.1 or greater
RN4020	Bluetooth LE	Transmit: 16 mA	Requires Windows 8.1 or greater

**Figure A1:**
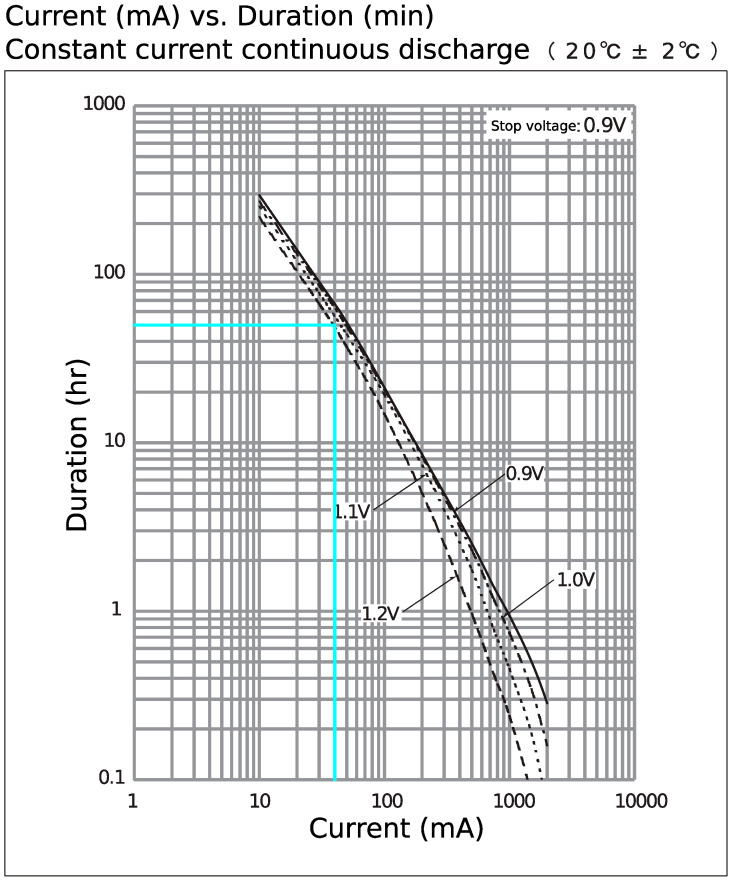
Battery operating time (lines were added to the original figure).

**Figure A2:**
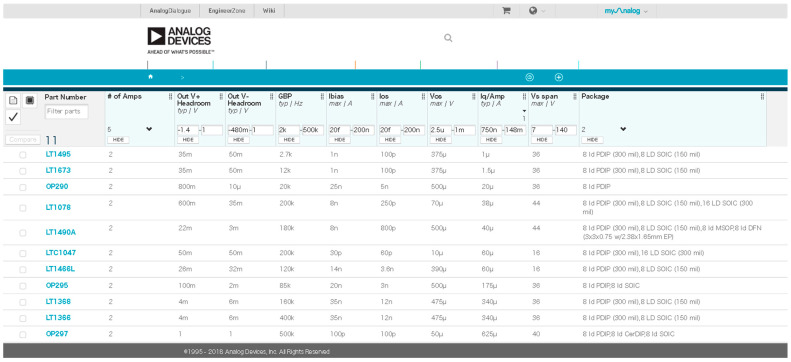
An example of search results for “op-amps” on the Analog Devices website.

**Figure A3:**
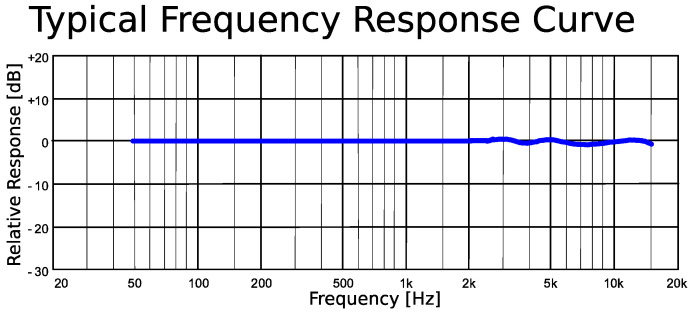
The frequency response of WM-61A from the datasheet. Frequency range: 20–16,000 Hz.

**Figure A4:**
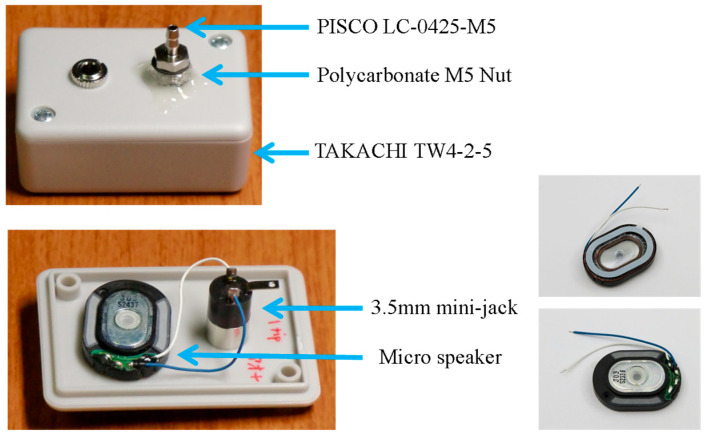
Micro-pressure generator.

## Data Availability

The data that support the findings of this study are available from the corresponding author, upon reasonable request.

## Appendix A

**Details of the equipment components.**

The case used was the LM-140C (Takachi Electric Industry, Saitama, Japan). The circuit used a universal board, and a LXDC55 (Murata Electronics, Kyoto, Japan)-based DCDC converter (3.3 V) was used to convert the voltage from four AA batteries to 3.3 V. A polypropylene plastic tube with an inner diameter of 6 mm was cut to 8 mm, an ECM was inserted, and a mini-fitting bulkhead connector (VFB226/VFBL10 (ISIS Co., Ltd., Osaka, Japan)) was connected to a 9 mm silicone tube with an inner diameter of 3 mm and an outer diameter of 6 mm. The nasal cannula was cut once at the left and right nostrils, filled with glue, and then reconnected to separate the left and right nostrils.

## Appendix B

The source code using Processing software to capture the measurement data.

Required packages:

Processing 3.5.4 (https://processing.org/, accessed on 18 May 2025);

controlP5 2.2.5 (https://github.com/sojamo/controlp5, accessed on 18 May 2025);

xbeeapi 0.9.3 (https://github.com/andrewrapp/xbee-api, accessed on 18 May 2025);

log4j 1.2.17 (https://logging.apache.org/log4j/1.x/, accessed on 18 May 2025);

RXTX 2.2pre2

(http://rxtx.qbang.org/wiki/index.php/Main_Page, accessed on 18 May 2025).

* *

import java.io.*;
import processing.serial.*;
import com.rapplogic.xbee.*;
import com.rapplogic.xbee.api.*;
import com.rapplogic.xbee.api.wpan.*;
import com.rapplogic.xbee.api.ZigBee.*;
import com.rapplogic.xbee.examples.*;
import com.rapplogic.xbee.examples.wpan.*;
import com.rapplogic.xbee.examples.ZigBee.*;
import com.rapplogic.xbee.socket.*;
import com.rapplogic.xbee.test.*;
import com.rapplogic.xbee.util.*;
* *
import controlP5.*;
ControlP5 cp5;
Chart adcChart;
* *
XBee xbee;
XBeeAddress16 sensor_address16 = new XBeeAddress16(0, 0);
* *
public void nodeDiscovery(int theValue) {
 if(xbee.isConnected() == false) { return; }
 println("nodeDiscovery");
 try{
  xbee.sendAsynchronous(new AtCommand("ND"));
 }
 catch(XBeeException xe){ ; }
 cp5.get(ScrollableList.class, "sensors").clear();
}
* *
public void setADC(int theValue) {
 if(sensor_address16.get16BitValue() == 0) { return; }
 println("setADC");
 XBeeAddress16 remoteAddress = sensor_address16;
 RemoteAtRequest request0 = new RemoteAtRequest(remoteAddress, "D0", 2);
 RemoteAtRequest request1 = new RemoteAtRequest(remoteAddress, "D1", 2);
 RemoteAtRequest request2 = new RemoteAtRequest(remoteAddress, "D2", 2);
 RemoteAtRequest request3 = new RemoteAtRequest(remoteAddress, "D3", 2);
 //int[] val = {0x13,0x88};
 int[] val = {0xff,0xfe};
 RemoteAtRequest request4 = new RemoteAtRequest(remoteAddress, "ST", val);
 try{
  xbee.sendAsynchronous(request0);
  xbee.sendAsynchronous(request1);
  //xbee.sendAsynchronous(request2);
  //xbee.sendAsynchronous(request3);
  xbee.sendAsynchronous(request4);
 }
 catch(XBeeException xe){ ; }
}
* *
public void startSampling(int theValue) {
 if(sensor_address16.get16BitValue() == 0) { return; }
 println("startSampling");
 XBeeAddress16 remoteAddress = sensor_address16;
 //int[] val = {0,0x64}; // = 100 ms, 10 Hz
 //int[] val = {0,0x50}; // = 80 ms, 12.5 Hz
 //int[] val = {0,0x4b}; // = 75 ms, 13.33 Hz
 int[] val = {0,0x32}; // = 50 ms, 20 Hz ~ actual 12.5 Hz
 RemoteAtRequest request0 = new RemoteAtRequest(remoteAddress, “IR”, val);
 try{
  xbee.sendAsynchronous(request0);
 }
 catch(XBeeException xe){ ; }
}
* *
public void stopSampling(int theValue) {
 if(sensor_address16.get16BitValue() == 0) { return; }
 println("stopSampling");
 XBeeAddress16 remoteAddress = sensor_address16;
 int[] val = {0,0};
 RemoteAtRequest request0 = new RemoteAtRequest(remoteAddress, "IR", val);
 try{
  xbee.sendAsynchronous(request0);
 }
 catch(XBeeException xe){ ; }
}
* *
void setup(){
 size(1024, 768);
 cp5 = new ControlP5(this);
 adcChart = cp5.addChart("ADC View")
        .setPosition(10, 10)
        .setSize(800, 400)
        .setRange(0, 1.2)
        .setView(Chart.LINE) // use Chart.LINE, Chart.PIE, Chart.AREA, Chart.BAR_CENTERED
        .setStrokeWeight(1.5)
        .setColorCaptionLabel(color(40));
* *
 adcChart.addDataSet("adc0");
 adcChart.setColors("adc0", color(200,50,0));
 adcChart.setData("adc0", new float[200]);
 adcChart.addDataSet("adc1");
 adcChart.setColors("adc1", color(0,200,100));
 adcChart.setData("adc1", new float[200]);
* *
 cp5.addButton("nodeDiscovery")
  .setValue(0)   .setPosition(220,440)   .setSize(200,20); 
 cp5.addButton("setADC")
  .setValue(0)   .setPosition(220,470)   .setSize(200,20); 
 cp5.addButton("startSampling")
  .setValue(0)   .setPosition(220,500)   .setSize(200,20); 
 cp5.addButton("stopSampling")
  .setValue(0)   .setPosition(220,530)   .setSize(200,20); 
* *
 cp5.addScrollableList("serialPort")
   .setPosition(10, 440)    .setSize(200,100)
   .setBarHeight(20)    .setItemHeight(20)
   .addItems(Serial.list())
   .setType(ScrollableList.LIST) // currently supported DROPDOWN and LIST
   ;
* *
 cp5.addScrollableList("sensors")
  .setPosition(430, 440)    .setSize(200,100)
  .setBarHeight(20)    .setItemHeight(20)
  .setType(ScrollableList.LIST) // currently supported DROPDOWN and LIST
  ;
* *
 cp5.addTextfield("outputfile")
  .setPosition(640, 440)
  .setSize(200,40)
  .setColor(color(255,0,0))
  ;
* *
 cp5.get(Textfield.class,"outputfile").setText("output.csv");
* *
xbee = new XBee();
}
* *
void serialPort(int n) {
 String port = (String)cp5.get(ScrollableList.class, "serialPort").getItem(n).get("text");
 print(port);
 if(xbee.isConnected() == true) {
  xbee.close();
 }
 try{
   xbee.open(port , 9600);
   System.out.print(" " + port + ">succeeded");
  }
  catch(XBeeException xe){
   System.out.print(" " + port + ">failed");
  }
 if(xbee.isConnected() == true) {
  xbee.addPacketListener(new XBeeListener());
 }
}
* *
public void controlEvent(ControlEvent theEvent) {
 println("controlEvent:" + theEvent.getController().getName());
}
* *
public static final int BUFSIZE = 200;
float[] adcbuf0 = new float[BUFSIZE];
float[] adcbuf1 = new float[BUFSIZE];
int ptbuf0 = 0;
int ptcount = 0;
* *
void draw() {
 background(140);
 if(ptcount != ptbuf0) {
  int diff = Math.abs(ptbuf0 - ptcount);
  try{
   Writer writer = new FileWriter(cp5.get(Textfield.class,"outputfile").getText(),true);
   if(diff < BUFSIZE/2) { // without loop back
    for(int i = ptcount;i < ptbuf0;i ++) {
     print("("+i+")");
     adcChart.push("adc0", adcbuf0[i]);
     adcChart.push("adc1", adcbuf1[i]);
     writer.write(nf(year(),4)+","+nf(month(),2)+","+nf(day(),2)+","+nf(hour(),2) + "," + nf(minute(),2) + "," + nf(second(),2) + ",");
     writer.write(String.format("%.3f",adcbuf0[i]) + "," + String.format("%.3f",adcbuf1[i])+"\r\n");
    }
   } else { // with loop end back
    for(int i = ptcount;i < BUFSIZE;i ++) {
     print("("+i+")");
     adcChart.push("adc0", adcbuf0[i]);
     adcChart.push("adc1", adcbuf1[i]);
     writer.write(nf(year(),4)+","+nf(month(),2)+","+nf(day(),2)+","+nf(hour(),2) + "," + nf(minute(),2) + "," + nf(second(),2) + ",");
     writer.write(String.format("%.3f",adcbuf0[i]) + "," + String.format("%.3f",adcbuf1[i])+"\r\n");
    }
    for(int i = 0;i < ptbuf0;i ++) {
     print("("+i+")");
     adcChart.push("adc0", adcbuf0[i]);
     adcChart.push("adc1", adcbuf1[i]);
     writer.write(nf(year(),4)+","+nf(month(),2)+","+nf(day(),2)+","+nf(hour(),2) + "," + nf(minute(),2) + "," + nf(second(),2) + ",");
     writer.write(String.format("%.3f",adcbuf0[i]) + "," + String.format("%.3f",adcbuf1[i])+"\r\n");
    }
   }
   writer.close();
  } catch(IOException ex) {
   ex.printStackTrace();
  } 
  ptcount = ptbuf0;
 }
}
* *
class XBeeListener implements PacketListener{
 public void processResponse(XBeeResponse response){
  ApiId id = response.getApiId();
  print(""+id.toString());
  switch(id){
   case ZNET_IO_SAMPLE_RESPONSE:
    ZNetRxIoSampleResponse ioSample = (ZNetRxIoSampleResponse)response;
    print(" AD0=" + String.format("%.3f", (float)ioSample.getAnalog0()*1.2/1024));
    print(" AD1=" + String.format("%.3f", (float)ioSample.getAnalog1()*1.2/1024));
    //print(" AD2=" + String.format("%.3f", (float)ioSample.getAnalog2()*1.2/1024));
    //print(" AD3=" + String.format("%.3f", (float)ioSample.getAnalog3()*1.2/1024));
    if(ptbuf0 >= BUFSIZE) { ptbuf0 = 0; }
    adcbuf0[ptbuf0] = (float)ioSample.getAnalog0()*1.2/1024.0;
    adcbuf1[ptbuf0] = (float)ioSample.getAnalog1()*1.2/1024.0;
    ptbuf0 ++;
    break;
   case ZNET_RX_RESPONSE:
    ZNetRxResponse rx = (ZNetRxResponse) response;
    println(ByteUtils.toBase16(rx.getRemoteAddress64().getAddress()));
    println(ByteUtils.toString(rx.getData()));
    break;
   case AT_RESPONSE:
    AtCommandResponse atResponse = (AtCommandResponse) response;
    if (atResponse.isOk()) {
     println("At Command successful: " + ByteUtils.toBase16(atResponse.getValue()));
     ZBNodeDiscover node = ZBNodeDiscover.parse(atResponse);
     println("NodeAddress16: " + ByteUtils.toBase16(node.getNodeAddress16().getAddress()));
     println("NodeAddress64: " + ByteUtils.toBase16(node.getNodeAddress64().getAddress()));
     cp5.get(ScrollableList.class, "sensors").addItem((String)ByteUtils.toBase16(node.getNodeAddress16().getAddress()),0);
     if(!sensor_address16.equals(node.getNodeAddress16()) && node.getNodeAddress16().get16BitValue() != 0) {
      sensor_address16 = node.getNodeAddress16();
      println("Sensor was registered.");
     }
    } else {
     println("At Command failed: " + ByteUtils.toBase16(atResponse.getValue()));
    }
    break;
   case REMOTE_AT_RESPONSE:
    RemoteAtResponse remoteAtResponse = (RemoteAtResponse) response;
    if (remoteAtResponse.isOk()) {
     println("RemoteAt Ok.");
    } else {
     println("RemoteAt Failed.");
    }
    break;
   default:
    print("?");
    break;
  }
  println(".");
 }
}

## Appendix C

Battery operating time (lines were added to the original figure).

https://jpn.faq.panasonic.com/app/answers/detail/a_id/29060/~/%5B%EF%BD%B1%EF%BE%99%EF%BD%B6%EF%BE%98%EF%BD%A5%EF%BE%8F%EF%BE%9D%EF%BD%B6%EF%BE%9E%EF%BE%9D%5D-%E4%B9%BE%E9%9B%BB%E6%B1%A0%E3%81%AE%E9%9B%BB%E6%B1%A0%E5%AE%B9%E9%87%8F%E3%81%AF%E3%81%A9%E3%82%8C%E4%BD%8D%EF%BC%9F-pz29060, accessed on 18 May 2025.
